# Atypical Features of *Thermus thermophilus* Succinate:Quinone Reductase

**DOI:** 10.1371/journal.pone.0053559

**Published:** 2013-01-07

**Authors:** Olga Kolaj-Robin, Mohamed R. Noor, Sarah R. O’Kane, Frauke Baymann, Tewfik Soulimane

**Affiliations:** 1 Chemical and Environmental Sciences Department and Materials & Surface Science Institute, University of Limerick, Limerick, Ireland; 2 Laboratoire de Bioénergétique et Ingénierie des Protéines, BIP/CNRS, UMR7281, AMU, Marseille, France; Griffith University, Australia

## Abstract

The *Thermus thermophilus* succinate:quinone reductase (SQR), serving as the respiratory complex II, has been homologously produced under the control of a constitutive promoter and subsequently purified. The detailed biochemical characterization of the resulting wild type (wt-rcII) and His-tagged (rcII-His_8_-SdhB and rcII-SdhB-His_6_) complex II variants showed the same properties as the native enzyme with respect to the subunit composition, redox cofactor content and sensitivity to the inhibitors malonate, oxaloacetate, 3-nitropropionic acid and nonyl-4-hydroxyquinoline-N-oxide (NQNO). The position of the His-tag determined whether the enzyme retained its native trimeric conformation or whether it was present in a monomeric form. Only the trimer exhibited positive cooperativity at high temperatures. The EPR signal of the [2Fe-2S] cluster was sensitive to the presence of substrate and showed an increased rhombicity in the presence of succinate in the native and in all recombinant forms of the enzyme. The detailed analysis of the shape of this signal as a function of pH, substrate concentration and in the presence of various inhibitors and quinones is presented, leading to a model for the molecular mechanism that underlies the influence of succinate on the rhombicity of the EPR signal of the proximal iron-sulfur cluster.

## Introduction

The succinate:quinone oxidoreductases (SQOR) superfamily (EC 1.3.5.1) comprises enzymes serving as the respiratory complex II and are classified depending on the direction of the reaction catalyzed *in vivo*. While the succinate:quinone reductases (SQRs) mediate the oxidation of succinate to fumarate coupled with the reduction of quinone to quinol, the quinol:fumarate reductases (QFRs) catalyze the reverse reaction [1]. SQR and QFR are homologous proteins that evolved from a common evolutionary ancestor and can catalyze both reactions *in vitro* and in the cell under the appropriate conditions [2], [3]. SQRs are involved in the aerobic metabolism and, as well as being a part of the respiratory chain, constitute the only membrane-bound enzyme of the tricarboxylic acid cycle [4]. In contrast, QFRs participate in anaerobic respiration with fumarate as the terminal electron acceptor [5], [6]. SQORs typically consist of three to four subunits: the hydrophilic subunits A (SdhA) and B (SdhB) containing a covalently-bound flavin cofactor and [2Fe-2S], [4Fe-4S], and [3Fe-4S] iron-sulfur clusters, respectively, and one large or two small membrane-bound subunits (C or C and D). The dicarboxylate oxidation/reduction and quinone reduction/oxidation sites are located in the subunit A and in the membrane anchor subunit(s), respectively. While the hydrophilic subunits are highly conserved among members of all domains of life, the sequence similarities between the membrane domains of complex II are significantly lower. SQORs are classified into five types (A–E) based on the number of membrane-bound domains and differences in the heme *b* composition. Enzymes with only one membrane subunit are grouped into type B as opposed to all the other types containing two hydrophobic domains. The heme content varies between zero (type D and E), one (type C) and two (types A and B). With the advent of the crystal structure of type D QFR from *Escherichia coli* more than a decade ago [7] followed by the structures of two prokaryotic and three mitochondrial SQORs [8], [9], [10], [11], [12], novel results have constantly emerged such as evidence for transmembrane proton transfer in di-heme QFR from *Wolinella succinogenes* [13]. Nonetheless, many questions are yet to be answered.

Previously, we have purified SQR from the extremely thermophilic bacterium *Thermus thermophilus* [14] and characterized it as a trimeric, di-heme, menaquinone (MK)-utilizing enzyme. The redox midpoint potential of its [3Fe-4S] center was determined to be at least 60 mV higher than that of its [2Fe-2S] center. This is in contrast to the hypothesis that MK-reducing SQRs are characterized by a higher midpoint potential for the [2Fe-2S] center with respect to the [3Fe-4S] center [1]. In addition, NQNO (nonyl-4-hydroxyquinoline-N-oxide), a semiquinone analog and inhibitor of quinone reactions in complex II, showed no influence on the redox behavior of the heme *b* moieties at variance with the equivalent *Bacillus subtilis* enzyme, where it was reported to induce a downshift of the midpoint potential of heme *b*_L_ [15] and a hysteresis in the titration behavior of both *b*-hemes [16]. Furthermore, characterization of *T. thermophilus* SQR revealed several novel features. These include the interprotomer temperature-dependent positive cooperativity in the trimeric complex as well as a modification of the EPR signal of the [2Fe-2S] iron-sulfur cluster, an immediate electron acceptor from the active site flavin, in the presence of the substrate succinate [14].

Most of the analyses on SQRs performed to date have involved the purification of native enzymes with the exception of organisms for which genetic manipulation techniques are well established, such as *E. coli* [9], *B. subtilis* [17], [18] or *Paracoccus denitrificans* [19]. Here we present a system for the recombinant expression of the *T. thermophilus* SQR and detailed characterizations of recombinant enzyme variants. Due to a modified purification procedure, expression of the recombinant form of the enzyme resulted in a higher quality complex II in larger quantities. This consequently allowed a more detailed biophysical characterization of the protein. The results presented herein represent a significant step towards the elucidation of a three-dimensional structure of type A SQOR, not available to date. Moreover, a detailed analysis of succinate influence in the active site on the shape of the EPR signal of the [2Fe-2S] cluster was also performed. Possible molecular bases of this phenomenon that may be of experimental use to monitor the occupancy of the active site of the enzyme by its substrate are discussed in this paper.

## Materials and Methods

### Recombinant Production of Complex II from *T. thermophilus* HB8

Vectors for homologous, recombinant production of complex II from *T. thermophilus* HB8 with polyhistidine tags on the C- or N-terminus of the SdhB subunit (rcII-SdhB-His_6_ and rcII-His_8_-SdhB, respectively) the and wild type, non-tagged (wt-rcII) forms of the protein were generated. For rcII-SdhB-His_6_, the entire *sdh* operon (gene loci TTHA1553-6) with an introduced His_6_ tag on C-terminus of the SdhB subunit was amplified using cII*For* (5′-atata*catatg* tacaggggaagcg aagggcagtgg-3′) and cII*RevHis* (5′-tatat*gcggccgc*ttaa**tgatgatgatgatgatg** gaagcggtccatgaggatcg cccgcttga-3′) primers and *Nde*I/*Not*I cloned into *E. coli/T. thermophilus* shuttle vector pDM12 [20] (a gift from Prof. Bernd Ludwig; Goethe Universität, Frankfurt, Germany) resulting in pDM12/rcII-SdhB-His_6_ vector.

The expression vector for rcII-His_8_-SdhB was constructed using an overlap PCR with cII*For* (above), His_8_SdhB_1*Rev* (5′-**GTGATGATGGTGATGATGGTGATG**catgcctccctcctagtag gtccgg-3′), His_8_SdhB_2*For* (5′-atg**CATCACCATCATCACCATCATCAC** caggtcacg ctgaaggtcctccgc-‘3) and cII*Rev* 5′-atatat*gcggccgc* ttagaagcggtccatgaggatcgc-3′) primers and the PCR product was cloned as above resulting in the pDM12/rcII-His_8_-SdhB vector.

In order to generate a vector for the expression of wild type, non-tagged form of the enzyme (wt-rcII), the entire *sdh* operon was amplified using cII*For* and cII*Rev* and cloned as above yielding the pDM12/wt-rcII vector. In the primer sequences restriction sites used for cloning are marked in *italics*, polyhistidine tag in **bold,** primer overlapping sequence is in uppercase and sequence complementary to the template is underlined. The resulted clones were sequenced prior to analysis.

For homologous expression, *T. thermophilus* HB8 was transformed with the generated expression vectors by electroporation and 50 mL liquid cultures were prepared from several colonies selected on 50 µg/mL kanamycin at 70°C in a water-saturated atmosphere. After an overnight growth in LB medium at 70°C, cell membranes were isolated and the expression clone was selected based on the most prominent signal judged by Western blot (His-tagged constructs) and on the absorbance at 558 nm in the dithionite reduced-*minus*-oxidized spectrum (wt-rcII).

The large-scale productions of all forms of the enzyme were performed in 5 L of LB supplemented with kanamycin at a final concentration of 50 µg/mL, at 70°C and 250 rpm for 24 h, yielding ∼ 30–40 g of wet biomass that was stored at –80°C until use.

### Isolation of Membranes

*T. thermophilus* cells were resuspended in 0.25 M Tris-HCl (pH 7.6) buffer containing 0.2 M KCl (Buffer A) in the ratio of 5 mL buffer to each gram of cell pellet and subsequently homogenized. Lysozyme was later added to a final concentration of 0.6 µM and the suspension was stirred for 3 h at room temperature. After centrifugation at 53936×*g* for 45 min at 4°C, the supernatant containing *T. thermophilus* soluble proteins was removed. The pelleted membranes were then resuspended in a small volume of Buffer A and protein concentration was analyzed using BCA Protein Assay Kit (Thermo Scientific Pierce) standardized against bovine serum albumin. Approximately 60 µg of membrane proteins was used in the subsequent SDS-PAGE and Western blotting analyses. For protein purification the membrane extract was diluted to a protein concentration of 10 mg/mL with the appropriate buffer and membrane proteins were solubilized by incubation for 3 h at 4°C in the presence of 5% Triton X-100 (Sigma Aldrich). Non-solubilized proteins were removed by centrifugation at 53936×*g* for 1 h at 4°C, yielding a clear membrane suspension.

### Purification of Recombinant Complex II from *T. thermophilus* HB8

Purification of wt-rcII was performed essentially as described for the native enzyme [14] with the omission of the first anion exchange purification step on DEAE-Biogel agarose (Biorad). All chromatographic steps were performed using the Äkta Prime or Äkta Explorer systems (GE Healthcare). The isolated membranes were solubilized in 0.01 M Tris-HCl, pH 7.6, 0.1% Triton X-100 and applied directly onto an XK 26/20 column, packed with 30 mL of Fractogel EMD TMAE (Merck) previously equilibrated with the same buffer. All further purification steps were performed as previously described [14].

Both His-tagged versions of the enzyme, rcII-SdhB-His_6_ and rcII-His_8_-SdhB, were purified according to the same protocol. Isolated membranes were solubilized in 0.05 mM Tris-HCl, pH 7.6, 300 mM NaCl, 10 mM imidazole and membrane proteins were solubilized with Triton X-100 as described above. The prepared extract was applied onto an XK 26/20 column, packed with 30 mL of Nickel Sepharose 6 Fast Flow resin (GE-Healthcare) pre-equilibrated with the same buffer. After sample binding to the resin, a detergent exchange step was performed by washing the column extensively with 0.05 M Tris-HCl, pH 7.6, 300 mM NaCl, 10 mM imidazole and 0.05% dodecyl-ß-D-maltoside (DDM) (Anatrace) until Triton X-100 has been removed from the sample, as determined from the 280 nm absorbance contributed by Triton X-100. Elution was performed with a linear gradient of 0 to 0.4 M imidazole, for 1 h at a flow rate of 1 mL/min. Complex II-rich fractions were pooled and diluted with 0.01 M Tris-HCl (pH 7.6), 0.05% DDM to reduce the conductivity to <2 mS/cm and subsequently applied onto an XK 26/20 column, packed with 30 mL of Fractogel EMD TMAE (Merck) previously equilibrated with 0.01 M Tris-HCl (pH 7.6), 0.05% DDM. Proteins were eluted with a linear gradient of 0 to 0.5 M NaCl, for 1 h at a flow rate of 2 mL/min. The fractions containing complex II were pooled and concentrated to 2 mL using a centrifugal filter (Vivaspin 20 MWCO 100 kDa, Sartorius) and applied onto a HiLoad XK 16/60 Superdex 200 gel filtration column (GE-Healthcare) previously equilibrated with 0.05 M Tris-HCl (pH 7.6), 0.05% DDM. The purified His-tagged complex II was concentrated to 10–15 mg/mL (Vivaspin 2 MWCO 100 kDa, Sartorius), aliquoted and snap frozen at −80°C.

Analytical SEC analyses of complex II were performed by applying 250 µg of purified protein onto a Superdex 200 10/300 GL gel filtration column (GE-Healthcare) previously equilibrated with 0.05 M Tris-HCl (pH 7.6), 0.05% DDM. The chromatography was carried out at the flow rate of 0.5 ml/min.

### Protein Analysis

Determination of protein concentrations, Blue Native PAGE analysis, redox titration, protein activation due to the bound endogenous oxaloacetate and subsequent activity measurements were performed as described previously [14]. In addition, the enzyme activity was assayed at 30°C in the presence of DCPIP (2,6-dichlorophenolindophenol), 0.05 mM nonyl-4-hydroxyquinoline-N-oxide (NQNO) and Vit. K_3_, DQ (duroquinone), 1,4-NQ (1,4-naphthoquinone) or *p*-BQ (*p*-benzoquinone) at concentrations of 0–1 mM.

Circular dichroism (CD) analysis was performed using a Chirascan CD spectrometer (Applied Photophysics) and Chirascan Pro-Data acquisition software. For the CD analysis in the far UV range (180 to 280 nm), quartz suprasil (QS) cuvettes of 0.1 mm path length (Hellma GmbH) were used. The baseline spectra and spectra for each protein sample were collected in triplicates with 4 s time points and 1 nm bandwidth. Baselines and protein spectra were separately averaged, and the averaged baselines were subtracted from the relevant averaged protein spectra and smoothed with the Savitsky-Golay algorithm. For thermal stability analyses, changes in the observed ellipticity at a single wavelength of 222 nm were analyzed in triplicates at increasing and subsequently decreasing temperatures in the 20–90°C range with 1°C ramp using quartz suprasil (QS) cuvettes of 10 mm path length (Hellma GmbH); the cuvette was covered with a lid to overcome the problem of rapid evaporation. Complex II was analyzed in 50 mM Tris-HCl (pH 7.6), 0.02% DDM at a concentration of 1 mg/mL (total volume 30 µL) for scans in the 180–280 nm range and 0.01 mg/mL (total volume 3.5 mL) for thermal analyses at 222 nm.

EPR spectra were recorded on a Bruker ElexSys X-band spectrometer fitted with an Oxford Instrument He-cryostat and temperature control system. Buffers used were either 50 mM MOPS at pH 7 or a mixture of 100 mM MOPS and 100 mM piperazine for the pH experiments. Samples were reduced by addition of 5 mM ascorbate, succinate (from a 1 M stock solution in water) or dithionite (from a 200 mM stock solution in 1 M MOPS buffer, pH 7). Additions of reducing agents were performed at room temperature followed by vortexing and freezing of the sample in liquid nitrogen within 5 min after addition. The addition of NQH_2_ and succinate in the presence of dithionite was done under argon. Protein concentration was 5–10 µM as indicated in the figure legends.

## Results and Discussion

### The Phenomenon of Positive Cooperativity

One of the novel features discovered during characterization of the *T. thermophilus* SQR was the interprotomer temperature-dependent positive cooperativity in the trimeric complex. As this behavior was not noted to date in other complexes II, to further confirm that the observed cooperativity is a genuine phenomenon and to provide valuable insights into the mechanism of action of a thermophilic SQR, a homologous expression system has been developed for this protein. In addition, existence of such a system will enable the future site-directed mutagenesis studies of this enzyme and it is expected to facilitate crystallogenesis and biophysical characterization of the protein by providing larger amounts of high quality enzyme through a simpler, reproducible purification protocol.

### Generation and Biochemical Analysis of Recombinant Complex II

**Figure 1:**
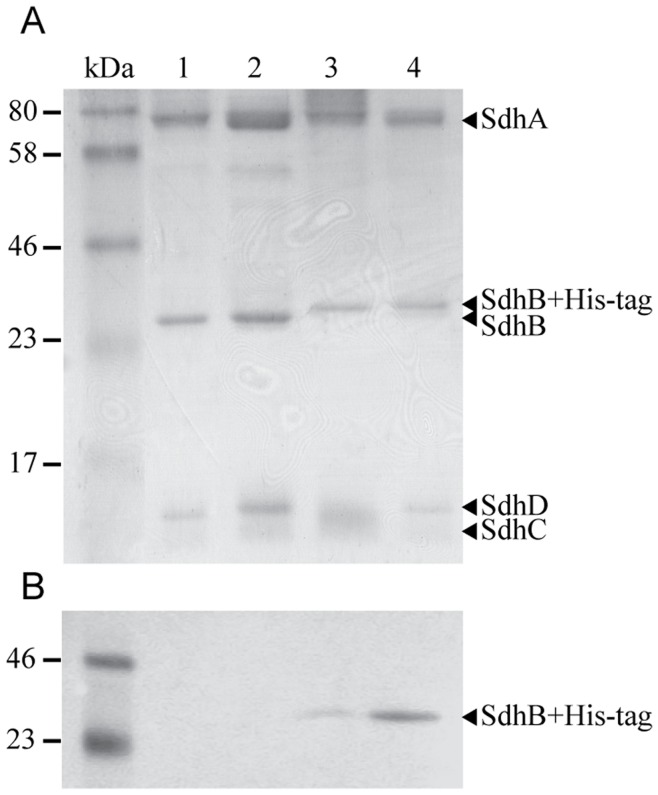
SDS-PAGE (A) and Western blot (B) analysis of purified complex II from *T. thermophilus.* Lanes: 1– native cII, 2– wt-rcII, 3– rcII-SdhB-His_6_, 4– rcII-His_8_-SdhB.

**Figure 2:**
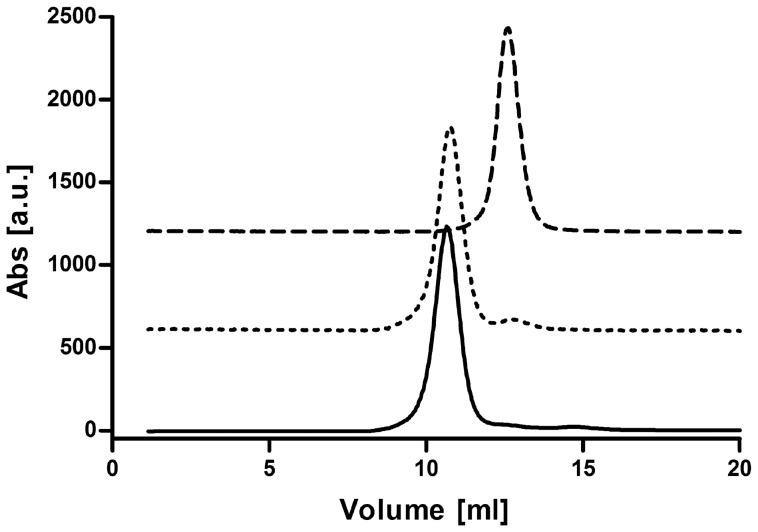
Stacked SEC elution profiles of wt-rcII (−), rcII-SdhB-His_6_ (−) and rcII-His_8_-SdhB (…).

Three recombinant variants of the enzyme were generated, all produced constitutively under the control of *bc* complex promoter from a *E. coli*/*T. thermophilus* shuttle vector [20] in the latter host. Expression clone for the production of the wild type complex II (wt-rcII) was selected based on the highest absorbance at 558 nm in the reduced-*minus*-oxidized spectrum of isolated membranes. The generated wt-rcII was purified to ∼95% homogeneity as described previously for the native enzyme [14] the with omission of the initial DEAE anion exchange chromatography. This purification procedure resulted in almost five-fold higher amounts of the protein in comparison to the native purification [14], corresponding to ∼6–8 copies of vector per cell, and yielded ∼37 mg of pure complex II from 100 g of *T. thermophilus* biomass. The existence of four subunits (SdhA, B, C and D) within the purified protein was confirmed by SDS-PAGE analysis (Fig. 1A, lane 1), while a single peak of Gaussian distribution after the final size exclusion chromatography demonstrates the sample homogeneity (Fig. 2). The heme *b* content in the purified sample was 16.43 nmol/mg of protein (Table S1), a value close to the theoretical content of 16.6 nmol/mg of protein, considering the molecular weight of the complex as 119.78 kDa and the existence of two heme moieties per protein monomer.

**Figure 3:**
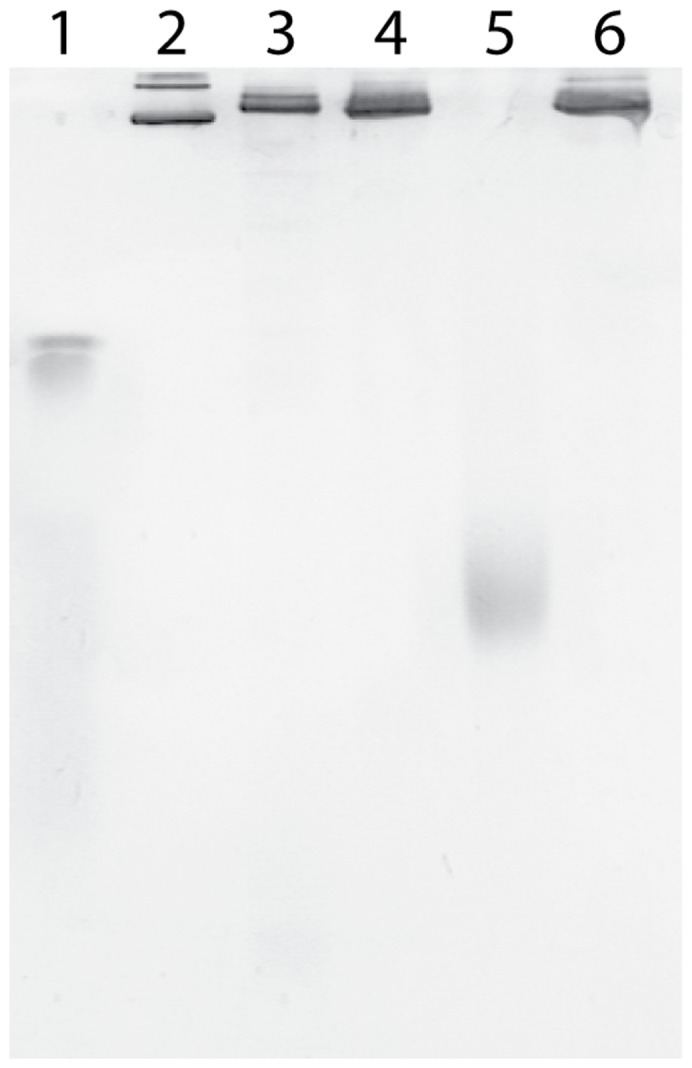
BN-PAGE analysis of purified complex II from *T. thermophilus.* Lanes: 1– β-amylase (200 kDa), 2– apoferritin monomer (443 kDa) and dimer (886 kDa), 3– native complex II from *T. thermophilus,* 4– wt-rcII, 5– rcII-SdhB-His_6_, 6– rcII-His_8_-SdhB.

The oligomerization state of the enzyme was evaluated by BN-PAGE (Blue Native PAGE) as described previously [14]. The wt-rcII sample shows a single band migrating in accordance to the native enzyme and indicates the trimeric nature of the protein, corresponding to ∼500 kDa considering 360 kDa from protein and an unknown contribution of detergent and lipid [14] (Fig. 3). Analysed by visible redox spectroscopy, the protein exhibited the same features as the native enzyme (Figure S1) while the existence of two heme *b* cofactors within wt-rcII was confirmed by optical Vis redox titration and midpoint potential values for hemes *b*_H_ and *b*_L_ were found to be in agreement with those determined for the native enzyme (Table S2). The presence of all redox cofactors of complex II, *i.e.* [2Fe-2S], [3Fe-4S] and [4Fe-4S] clusters, heme *b* and flavin was confirmed by EPR spectroscopy. Similar to the native enzyme [14], the rhombicity of the signal of the [2Fe-2S] center was dependent on the reductant used and higher in the presence of substrate succinate than after reduction with ascorbate or dithionite (see below).

Having confirmed that the developed homologous expression system provides recombinant SQR of characteristics identical to the native enzyme, an artificial monomeric version of the enzyme was generated. Since the cooperativity is strictly related to multiple binding sites within a protein, and, hence, in the case of complex II, to its trimeric nature, it is expected that it will be abolished in the artificial monomeric version of the enzyme. Based on the generated homology model of *T. thermophilus* complex II, we have identified the C-terminus of the iron-sulfur subunit (SdhB) as the ideal location for protein engineering to disrupt the oligomerization (Fig. S2). In addition, to combine the generation of a monomeric complex II with the simplification of protein purification, a hexahistidine affinity tag on the aforementioned C-terminus of the iron-sulfur subunit has been introduced.

A derivative of *E. coli/T. thermophilus* shuttle vector [20] encoding the His-tagged version of complex II (rcII-SdhB-His_6_) was prepared and the clone exhibiting the highest expression levels was selected based on the signal of the SdhB-His_6_ subunit detected by Western blotting in isolated membranes. The enzyme was purified sequentially through nickel immobilized metal affinity chromatography, TMAE anion exchange and size exclusion chromatography steps and yielded ∼35 mg of the ∼98% pure rcII-SdhB-His_6_ from 100 g of *T. thermophilus* biomass with the content of heme *b* in the purified sample of 16.6 nmol/mg of protein, (Fig. 1, lane 3; Table S1). The rcII-SdhB-His_6_ reproducibly eluted later in the gel filtration chromatography compared to the native and wt-rcII complexes (Fig. 2). While this fact was an early indication of the successful creation of a monomeric recombinant complex II, the purified rcII-SdhB-His_6_ was subjected to BN-PAGE to further evaluate whether the positioning of a His-tag on the C-terminus of SdhB subunit affected the oligomeric state of the protein. The band corresponding to rcII-SdhB-His_6_ (Fig. 3) migrates significantly faster than the trimeric native and wt-rcII as well as β-amylase (200 kDa). Therefore, we concluded that trimerization was disrupted in rcII-SdhB-His_6_ due to the insertion of a hexahistidine tag on the C-terminus of SdhB subunit and the resulting protein complex could only form a monomer.

In order to verify the existence of the positive cooperativity effect in the recombinantly produced, tagged enzyme, another His-tagged version of the enzyme has been generated. The major challenge was to choose an ideal location for the placement of the affinity tag that retains native complex oligomeric state. Furthermore, the length of His residues was increased from six to eight to increase the probability of a sufficient exposure of the tag in the folded complex and ensure a tight binding of the latter to the Ni-Sepharose resin. This approach has been employed successfully for a number of proteins including membrane-bound receptors [21], [22]. A careful place for tag insertion is crucial as it may not only abolish the native oligomeric state of the protein but may also promote a non-native oligomerization [23]. An insertion within the linear protein sequence was judged to be less preferable than an insertion at a terminal location of each subunit. Therefore, we identified the N-terminus of SdhB subunit as an appropriate site for the affinity tag. A derivative of *E. coli/T. thermophilus* shuttle vector [20] encoding the rcII-His_8_-SdhB was prepared and the enzyme was generated and purified as described above for the rcII-SdhB-His_6_. This yielded ∼35 mg of the ∼98% pure rcII-His_8_-SdhB from 100 g of *T. thermophilus* biomass with the heme *b* content in the purified sample of 16.6 nmol/mg of protein (Table S1). Placing the affinity tag on the N-terminus of the SdhB subunit did not affect the native trimeric state as demonstrated on BN-PAGE gel (Fig. 3).

On SDS-PAGE all four subunits of the rcII-His_8_-SdhB complex were detected, with a doublet for the band corresponding to SdhB (Fig. 1A, lane 4). While the upper band of this doublet represents the His-tagged SdhB subunit (Fig. 1B), the lower band migrates identically to the SdhB subunits of the native and wt-rcII complex II and therefore most likely corresponds to a native, non-tagged SdhB subunit. The addition of fumarate, the end product of the reaction catalyzed by SQR, at a concentration up to 50 mM [24] did not inhibit the expression of the native complex II as obtained total yields of the rcII-His_8_-SdhB as well as the ratio between the untagged and His-tagged SdhB subunit remained unchanged. Therefore, due to the trimeric nature of the protein, the formation of the complexes containing mixed, His-tagged and untagged SdhB species is not surprising. As expected, due to enhanced expression of the His-tagged complex and the affinity method of purification, the His_8_-SdhB comprises a significant majority of the observed species in SdhB hybrid. An obvious way to overcome this problem and to enable mutagenesis studies would be the overexpression of the enzyme in a *T. thermophilus* strain where background expression of any enzymes capable of succinate oxidase activity is eliminated. Although complex II deletion strains of *E. coli* [25] and *B. subtilis* [17] exist, despite our best efforts, the trials to generate *sdhCDAB* deletion strain of *T. thermophilus* have been unsuccessful so far. The ability to generate the monomeric version of the enzyme, subsequent tag cleavage and *in vitro* trimerization of the complex II may seem to be a solution to overcome the problem of mosaic SdhB composition and for future generation of mutants. We fear, however, that the amino acid residues remaining on the C-terminus of SdhB subunit after His-tag cleavage would still impede trimer formation due to the sensitive location of the affinity tag (Figure S2).

Both rcII-SdhB-His_6_ and rcII-His_8_-SdhB exhibited the same features as the native and wt-rcII enzymes as determined by visible redox spectroscopy. The presence of all redox cofactors of complex II was confirmed by EPR spectroscopy and the higher rhombicity of the signal of the [2Fe-2S] center induced by the presence of succinate was also observed. Optical redox titration confirmed the existence of two heme *b* cofactors with the midpoint potentials of *b*_H_ and *b*_L_ in agreement with both native and wt-rcII (Table S2).

**Figure 4:**
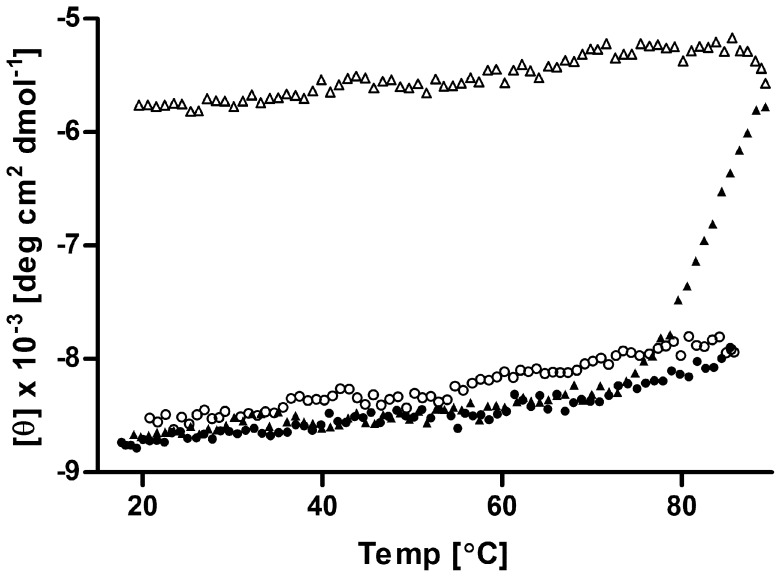
Circular dichroism stability analysis of recombinant complex II from *T. thermophilus.* The figure shows the dichroic activity at 222 nm of rcII-His_6_-SdhB (triangles) and rcII-SdhB-His_8_ (circles) recorded at increasing (▴, •) and subsequently decreasing (Δ, ○) temperature.

Analyzed by CD spectroscopy, all recombinant variants of *T. thermophilus* complex II exhibit bands characteristic for both predominantly-helical soluble and membrane proteins with twin negative bands at ∼222 and ∼210 nm and a positive band at ∼192 nm [26]; this is identical to the native complex II [14]. Although it is not possible to generate typical thermal unfolding curves for those highly thermostable enzymes due to hardware limitations, several conclusions can be drawn from the plot of the observed ellipticity at 222 nm to temperature profiles obtained for all recombinant versions of complex II (Fig. 4). Since the temperature profiles at 222 nm were identical for wt-rcII and rcII-His_8_-SdhB, only the results for rcII-His_8_-SdhB and rcII-SdhB-His_6_ are presented for clarity. The recombinant trimeric versions of complex II, wt-rcII and rcII-His_8_-SdhB exhibit a rather constant ellipticity at 222 nm throughout the temperature range tested, with only a slight difference observed between 20 and ∼90°C. Moreover, this change was almost completely reversible in the presented setup (Fig. 4). Compared to the native enzyme [14], the wt-rcII and rcII-His_8_-SdhB show a significantly higher thermostability. This may possibly be attributed to the much shorter purification procedure and a more limited contact with the relatively harsh detergent Triton X-100– a consequence of higher protein expression level that could prevent protein delipidation. In contrast, monomeric rcII-SdhB-His_6_ exhibited a significantly lowered, almost completely irreversible thermostability profile (Fig. 4). Although adopting higher oligomerization states is one of the evolutionary strategies to attain a higher thermostability [27], we cannot consider it as the case for complex II as many of the homologous enzymes from mesophilic prokaryotes also exist as trimers [9], [28]. Undoubtedly, however, the disruption of the trimeric nature of the protein negatively affects its stability, as one would expect.

Similar to the native protein [14], the recombinant untagged variant has also been crystallized with the maximum crystal size being only about half that of the native version. Given the increased purity of the recombinant protein, this is a perplexing observation. A preliminary screening of the crystals resulted in an anisotropic diffraction to 3.8 Å in the best direction using synchrotron radiation in contrast to the maximum of 8 Å with native protein crystals [14] using home source. Presumably, the smaller recombinant crystals froze better than the larger crystals of native complex. As this resolution is judged to be too low for biological interpretation, even without considering the possible data truncation due to the anisotropy, we are currently attempting further optimizations. It is hoped that more rapid purification procedure of rcII-His_8_-SdhB and its higher thermostability may further improve the crystals.

### Kinetic Analysis of Recombinant Complex II

The succinate dehydrogenase activity of the recombinant versions of complex II was measured using solution assays with PMS (phenazine methosulfate) as the intermediate and DCPIP as the terminal electron acceptor. Similar to the native complex II, all the recombinant variants of the enzyme required activation before activity assays could be conducted, a fact well known for complex II preparations and attributed to the presence of oxaloacetate in the active site [14]. The results summarizing the steady-state kinetic analysis of the complex II are presented in Table 1 and they show that all forms of the enzyme exhibit classical Michaelis-Menten kinetics at 30°C with the K_M_ values for the recombinant complex II in the range of 0.33–0.39 mM, reasonably in agreement with the native enzyme (0.21 mM). The turnover numbers (*k_cat_*) for the recombinant forms of the enzyme are up to two fold higher in comparison to the native complex II (Table 1). This higher specific activity is rather less likely to be related to the enzyme purity which is very comparable between the generated variants of complex II; instead, it is an effect of enzyme stability in the recombinant samples. Indeed, the specific activity expressed by the turnover number is lower for the native enzyme and the monomeric rcII-SdhB-His_6,_ which is reflected in their limited stability in comparison to the trimeric recombinant forms of the complex (see above). At higher temperature (70°C), both trimeric wt-rcII and rcII-His_8_-SdhB exhibit positive cooperativity as observed previously for the native complex II with k’ ranging between 0.25 and 0.28 mM (native enzyme k’ = 0.39 mM). The calculated Hill coefficients *h* for these enzymes are remarkably similar (Table 1) and indicate a minimum, and most likely the actual number of three substrate binding sites on the oligomeric enzyme with one site per protomer. As mentioned before, it was anticipated that the positive cooperativity observed for the native complex II at high temperatures will not be observed in the artificially produced monomeric rcII-SdhB-His_6_ version of the enzyme due to the lack of multiple active sites within the protein. Indeed, rcII-SdhB-His_6_ shows a standard Michaelis-Menten kinetics at 70°C with an unchanged affinity for the substrate in comparison to 30°C (K_M, 70°C_ = 0.29 mM *vs.* K_M, 30°C_ = 0.33 mM) and a more than three-fold increase in turnover number, typical at higher temperatures. The generation of the monomeric complex II and the obtained steady-state kinetic measurements undoubtedly confirm the existence of cooperatively interacting active sites within the trimeric complex II at higher temperatures and establish the *T. thermophilus* complex II as the first SQOR with such a kinetic behavior. It is difficult to predict how the disruption of the native oligomeric state of the enzyme will affect its activity as in some cases the existence of one particular oligomeric state is absolutely essential to retain activity of an enzyme [29], [30], [31] while generation of non-native oligomeric forms showing enhanced activity has also been reported [32]. For the complex II, our results clearly show that trimerization is not a prerequisite factor for the activity of the enzyme. Although based on edge-to-edge distances between the redox centers it has been suggested that electron transfer in SQR from *E. coli* likely occurs within each protomer rather than between adjacent ones [9], to the best of our knowledge, this report is the first one that confirms this theory.

**Table 1:** Summary of succinate dehydrogenase activity of complex II from *T. thermophilus.*

	Native complex II	wt-rcII	rcII-SdhB-His_6_	rcII-His_8_-SdhB
**Kinetics at 30°C**	K_M_ = 0.21±0.01 mM	K_M_ = 0.36±0.01 mM	K_M_ = 0.33±0.01 mM	K_M_ = 0.39±0.03 mM
	*k_cat_* = 500±10 min^−1^	*k_cat_* = 1020±4 min^−1^	*k_cat_* = 760±6 min^−1^	*k_cat_* = 890±16 min^−1^
**Kinetics at 70°C**	k’ = 0.39±0.08 mM	k’ = 0.25±0.03 mM	K_M_ = 0.29±0.02 mM	k’ = 0.28±0.04 mM
	*h* = 2.105±0.183	*h* = 2.285±0.128	*k_cat_* = 2500±31 min^−1^	*h* = 2.698±0.204
**Malonate inhibition**	cK_i_ = 40±4 µM	cK_i_ = 65±8 µM	cK_i_ = 58±4 µM	cK_i_ = 55±5 µM
**Oxaloacetate inhibition**	cK_i_ = 17±2 µM	cK_i_ = 51±3 µM	cK_i_ = 21±2 µM	cK_i_ = 85±12 µM
**3-NP inhibition**	iK_iapp_ = 0.23±0.06 mM	iK_iapp_ = 0.2±0.02 mM	iK_iapp_ = 0.29±0.06 mM	iK_iapp_ = 0.27±0.07 mM
**NQNO inhibition** *	nK_i_ = 70±5 µM	nK_i_ = 88±6 µM	nK_i_ = 80±8 µM	nK_i_ = 77±8 µM

c competitive inhibition.

i irreversible inhibition.

n non-competitive inhibition.

* measured in the DCPIP/1,4-NQ set up; all other measurements performed in the PMS/DCPIP set up.

As previously investigated with the native enzyme, the influence of standard inhibitors targeting the active site [malonate, oxaloacetate and 3-NP (3-Nitropropionic acid)] on the succinate dehydrogenase activity of the wt-rcII, rcII-SdhB-His_6_ and rcII-His_8_-SdhB was tested using the PMS/DCPIP assay at 30°C (Table 1). Although the obtained K_i_ values, in particular for oxaloacetate, suggest a slightly altered sensitivity of some recombinant versions of the enzyme when compared to the native one, they remained within the same order of magnitude and therefore can be considered as similar. Moreover, the addition of 1,4-naphthoquinone (1,4-NQ) as a direct electron acceptor increased the succinate dehydrogenase activity by a factor of two in comparison to when DCPIP was used as the only electron acceptor for all forms of the protein. The presence of *p*-BQ in the DCPIP assay induced a lag in the reduction of DCPIP the time of which was proportional to the *p*-BQ concentration. This directly confirms our previous published interpretation of the action of *p*-BQ that indeed acts as an electron acceptor of complex II and accumulates electrons due to its high redox midpoint potential [14]. Only once the *p*-BQ pool is largely reduced the electrons can proceed to DCPIP.

NQNO, a semiquinone analog, has a negligible effect on the DCPIP/PMS activity as expected based on the previous reports [1], [14], [28], [33]. It inhibited the DCPIP/1,4NQ and the DCPIP only activity to 40% and 70%, respectively, in a non-competitive mode with a K_i_ in the range of 70–88 µM throughout all the generated variants of the complex II (Table 1). This is further discussed in the EPR section below.

### EPR Signature of the [2Fe-2S] Cluster

In the preceding paper [14], we reported that the rhombicity of the EPR signal of the [2Fe-2S] cluster from the native complex II from *T. thermophilus* was dependent on the reductant used in the assay. The signal appeared axial with a g_x_ at 2.02 and a g_y/z_ at 1.926 if the sample has been reduced by ascorbate but rhombic with a g_x_ at 2.027, a g_y_ at 1.927 and a g_z_ at 1.912 if succinate was the electron donor. The same phenomenon has been observed with all three constructs of recombinant complex II and therefore its detailed characterization has been performed in this work.

To the best of our knowledge, the change in the rhombicity of the EPR signal of the [2Fe-2S] cluster under different experimental conditions was reported only by Beinert and coworkers on mitochondrial complex II [34]. They observed increasing rhombicity of the EPR signal in the course of a dithionite titration. In the case of *T. thermophilus* complex II, a similar but less pronounced increase in the rhombicity (g_x_ at 2.022 and a g_y/z_ at 1.926) was observed when 100-fold excess of dithionite was added to the enzyme (Figure S3). In both cases, the resulting signal is of a much smaller rhombicity than the signal that has been observed for the enzyme in the presence of succinate.

**Figure 5:**
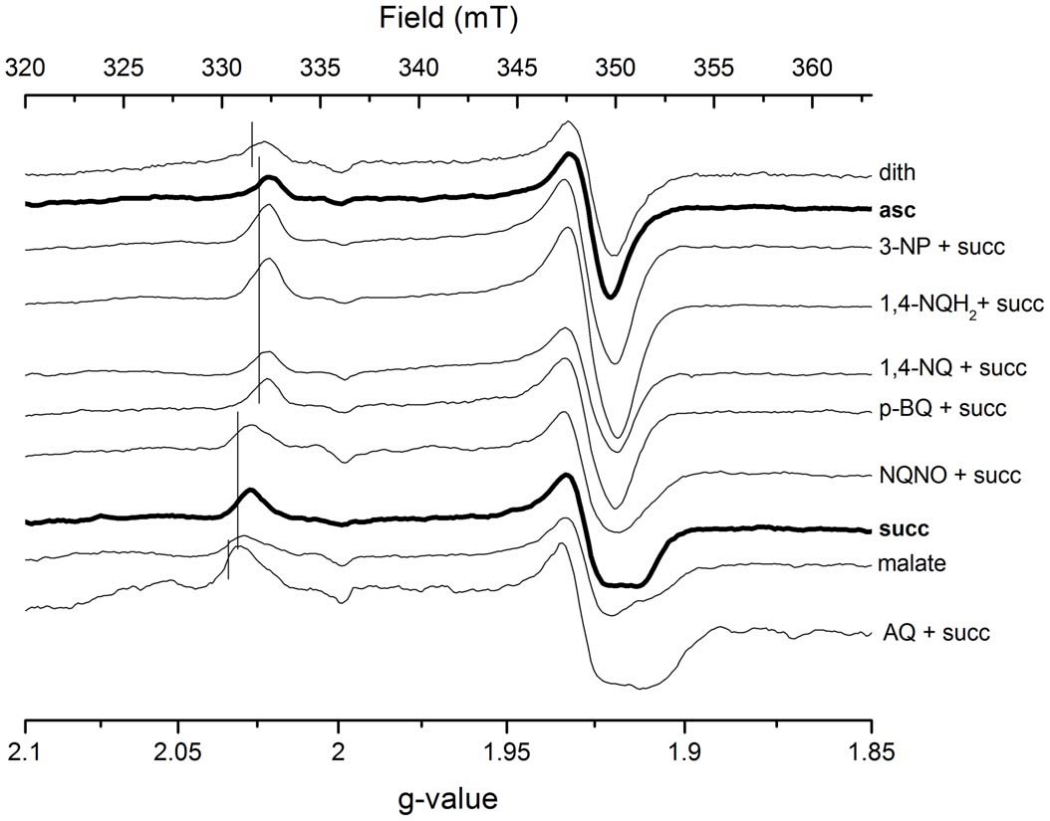
Influence of redox mediators, buffer and inhibitors on EPR spectra of the recombinant complex II. The rcII (5 µM) was reduced by the addition of 20 mM succinate followed by the addition of *p*-BQ, 1,4-NQ, 1,4-NQH_2_, AQ (100 µM each), as well as 3-NP (30 µM) and NQNO (700 µM). The spectrum labeled ‘asc’ was obtained on a sample reduced by 5 mM ascorbate, ‘malate’ by addition of 20 mM malate, ‘dith’ by addition of 1 mM dithionite. Vertical lines indicate the position of the g_x_-signal. Spectra were recorded at a temperature of 50 K, microwave power of 64 mW and a modulation amplitude of 1 mT.

**Figure 6:**
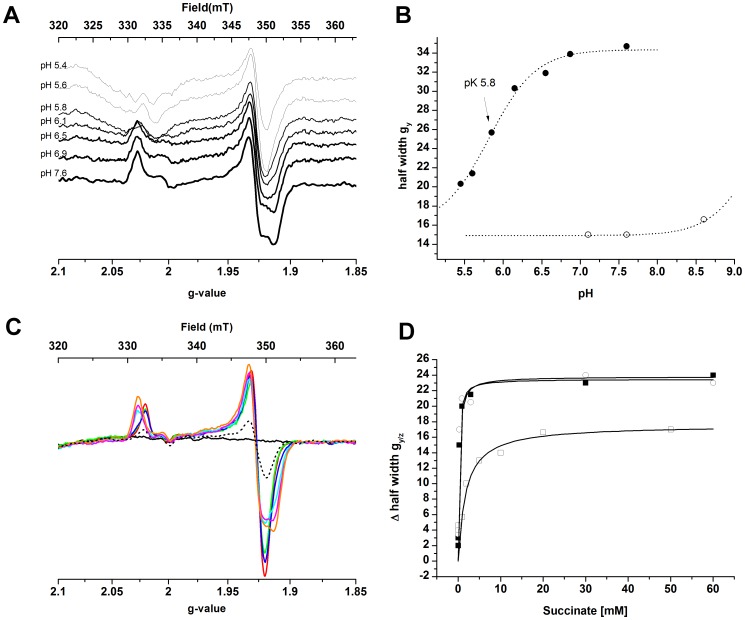
Influence of pH and succinate concentration on the EPR spectra of the recombinant complex II. The influence of pH and succinate concentration on the recombinant complex II EPR spectra are presented in panels A&B and C&D, respectively. **A** - EPR spectra of recombinant complex II (6 µM protein) in the presence of 40 mM succinate at the pH-values indicated (MOPS/Piperazine buffer 100 mM each); **B** – half width of the *g*_y/z_ signal of the [2Fe-2S] cluster of succinate- (•) and ascorbate-reduced (○) recombinant complex II. **C** - EPR spectra of the [2Fe-2S] cluster in the presence of 0 mM (black) 0.03 mM (red), 0.06 mM (green), 0.3 mM (blue), 3 mM (cyan), 30 mM (magenta) and 60 mM (orange) succinate. The dashed line represents the spectrum recorded after passing the sample with 60 mM succinate over a desalting column to remove succinate; **D** – The increase in half width of the g_y/z_ signal as a function of the succinate concentration with respect to the signal of the ascorbate-reduced sample (▪) was fitted with a K_M_ of 0.22 mM. Pre-reduction of the sample by an excess of dithionite (□) resulted in a K_M_ of 1.7 mM. Protein concentration was 10 µM. Spectra were recorded at a temperature of 50 K, microwave power of 64 mW and a modulation amplitude of 1 mT.

To further investigate the succinate effect, we probed EPR spectra of the [2Fe-2S] center in the presence of the inhibitors malate, 3-NP and NQNO and various quinones and mediators (Fig. 5), at different pH values (Fig. 6A–B) and in the presence of different amounts of succinate (Fig. 6C–D). The amount of succinate necessary to induce the transition from the axial to the rhombic EPR signal of the [2Fe-2S] signal was in the hundreds of micromolar, close to the K_M_ of succinate determined by activity assays (Table 1; the K_M_ value determined by activity measurements reflects the presence of succinate in the active site if the electron transfer from succinate to DCPIP is slower than the dissociation of product fumarate from the active site). In the presence of 40 mM succinate, a pK value of 5.8 was determined for the transition from the axial to the rhombic EPR signal of the [2Fe-2S] cluster (Fig. 6B). The suicide inhibitor 3-NP that covalently binds to the active site [10] prevented the transition to the rhombic signal when succinate was added, whereas malate could induce the rhombic signal in the absence of succinate (Fig. 5). NQNO, a semiquinone analog that inhibits complex II activity [1] had no significant influence on the [2Fe-2S] signal. If the flavin in the active site was pre-reduced by addition of an excess of dithionite, three times the concentration of succinate was necessary to induce the rhombic signal, in agreement with literature values on the influence of the redox state of the active site on the K_M_ of succinate [35], [36]. Taken together, these results suggest that succinate or the chemically similar malate, localized in the active site, have an influence on the rhombicity of the [2Fe-2S] EPR signal.

**Figure 7:**
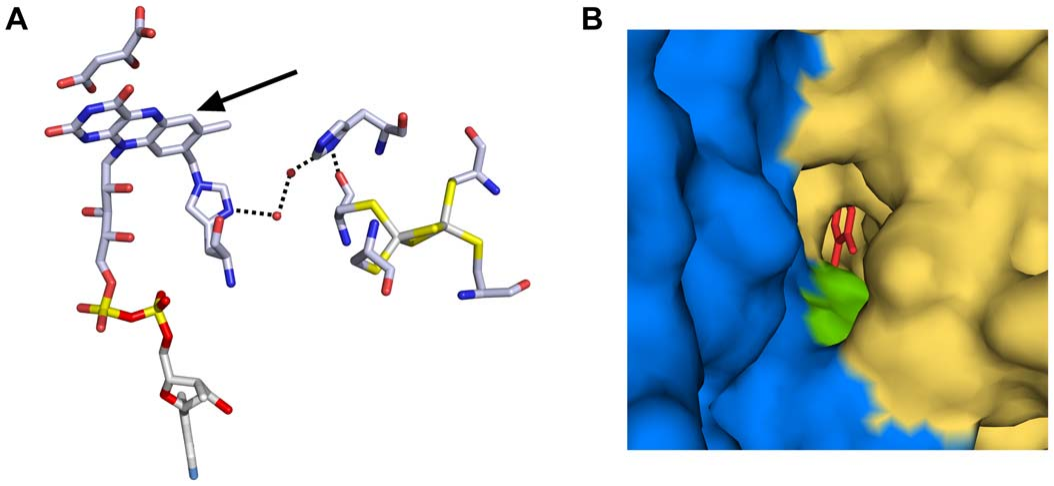
H-bond network between the flavin and the [2Fe-2S] cluster in complex II. The active site of the succinate dehydrogenase from *Gallus gallus* with 3-NP bound (PDB ID: 1YQ4) where Glu 67 from the iron-sulfur subunit was replaced by His is presented in panel A. An H-bond network connects one of the Cys ligand of the [2Fe-2S] cluster to the histidine that covalently links the flavin *via* two water molecules and a His residue. The arrow indicates the position of the cavity leading from the protein suface to the active site; Surface representation of the surroundings of the cavity leading to the active site is presented in panel B. Iron-sulfur subunit and flavin subunit are presented in blue and yellow, respectively. The His residue in the place of Glu 67 is shown in green; van der Waals ratios of O, N and C were set to 60, 65 and 70 respectively. The stick model represents the flavin cofactor.

Changes in the rhombicity of the EPR signal of [2Fe-2S] clusters have been attributed to a change in the angle between the iron/iron plane of the cluster and the plane between the sulfur ligand and its adjacent carbon atom [37], therefore implying a structural rearrangement of the cluster environment. Some of the several available 3D structures of complexes II resolve the position of water molecules and show several of them at the interface between subunits A and B close to the flavin and the [2Fe-2S] cluster, connecting the two cofactors *via* a hydrogen bond network. Figure 7A shows the structure of SQR from *Gallus gallus* with 3-NP bound. The glutamic acid residue at position 67 of the iron-sulfur subunit (SdhB-E67^Gg^) which is a part of the CxExxCGxC motif ligating the [2Fe-2S] cluster, has been replaced by a histidine that occupies the equivalent position in *T. thermophilus* (SdhB-H56^Tth^) according to multiple sequence alignments (Fig. S4). If an interaction between the substrate in the active site and the [2Fe-2S] cluster indeed occurs *via* the H-bond network, the presence of a His residue in this network may result in the pK of 5.8 that we observed for the appearance of the succinate-induced rhombic EPR signal. Figure 7B shows access to the active site of *Gallus gallus* SQR that is buried inside the protein. From the surface of the enzyme a cavity that is situated at the interface between the flavin and the Fe-S cluster subunits leads to the active site. The His residue that replaces Glu67 from *G. gallus* surfaces within this cavity.

The expected structural changes that influence rhombicity of the [2Fe-2S] cluster EPR signal are too small to be detected by X-ray crystallographic analysis, unless at ultra-high resolution (better than 1 Å) and may not have any impact on the function of the enzyme. They are, however, a precious experimental probe to assess the occupancy of the active site. As an example, different quinones, generally used as mediators in redox titrations, prevented transition from the axial to the rhombic EPR signal when succinate was added (Fig. 5). The addition of these quinones to a succinate-reduced sample induced transition of the signal back to its axial form. Small concentrations at 20 µM *p*-BQ and 10 µM 1,4-NQ thus were sufficient to obtain the axial signal in the presence of 20 mM succinate from a 6 µM rcII sample. The 1,4-NQH_2_ had the same effect as 1,4-NQ. Antraquinones (AQ), however, induced an increased rhombicity of the [2Fe-2S] signal in a succinate-reduced sample. We do not know at present why these quinones act differently on the [2Fe-2S] EPR signal but we postulate that the fact that they disturb the effect of succinate on the [2Fe-2S] signal indicates that they reach a position in or close to the enzyme active site. Kinetic experiments with NQNO support this interpretation. Available data (reviewed in [1]) have been interpreted as NQNO blocking a quinone binding site on the membrane anchor subunit(s) of complex II. In the activity assays of *T. thermophilus* complex II, the addition of NQNO partially inhibits enzyme activity in a non-competitive manner, indicating that mediators can accept electrons from one of the cofactors of the enzyme outside the quinone binding site. PMS/DCPIP activity assays are inhibited to only 5% by NQNO, whereas DCPIP-only activity is inhibited to 70%, translating the capacity of the small PMS molecule to more efficiently access complex II cofactors. DCPIP/1,4-NQ activity is inhibited to 40% by NQNO, showing that 1,4-NQ, just as PMS, can accept electrons from complex II elsewhere than from the quinone binding site blocked by NQNO. Since we discovered that 1,4-NQ perturbs the succinate-induced rhombicity of the [2Fe-2S] signal, it may interact with the active site and not only with the membrane anchor subunit as proposed in our previous report [14]. This result is certainly of no physiological relevance since the quinones present in the membrane of organisms should not reach the active site. It signifies, however, that caution has to be taken when studying the interaction of exogenous quinones with complex II in activity assays under the assumption that they interact exclusively with the quinone binding site of the complex.

### Concluding Remarks

The homologous production of the complex II from *T. thermophilus* under the control of the *bc* complex promoter presented herein made possible an efficient preparation method of the enzyme for structural and functional studies. By placing an affinity tag on different positions of the SdhB subunit, as guided by the examination of crystal structures of the enzyme from other organisms, trimeric as well as monomeric forms of the enzyme were successfully created. Only the trimeric forms exhibited cooperativity at high temperatures. This provided the experimental evidence that this novel feature of SQORs reported previously for the native *T. thermophilus* complex II is a genuine phenomenon of the trimeric enzyme and that each protomer is indeed involved in succinate oxidation.

All recombinant forms of the enzyme exhibits an EPR signal of the [2Fe-2S] cluster, the rhombicity of which depends on the presence of succinate or its analog malate in the active site. Mediators, such as quinones, interfered with this effect, indicating that in our experiments these molecules interact with the active site flavin and/or the [2Fe-2S] cluster and bind not only to the quinone binding sites as thought previously. Rhombicity of the [2Fe-2S] signal of *T. thermophilus* complex II thus provides a specific probe for occupancy of the active site and may, therefore, be of experimental use to address the molecular mechanism of succinate binding and primary electron transfer events in this enzyme. We propose a model for the molecular basis of the succinate influence on the geometry of the proximal iron-sulfur cluster ligand environment based on the available SQOR crystal structures and on sequence information.

## Supporting Information

Figure S1 **VIS spectra of recombinant complex II from** ***T. thermophilus*** **in its oxidized (black), succinate-reduced (red) and dithionite-reduced state (blue).** All the recombinant variants of complex II exhibited the same spectroscopic features as the native enzyme with absorption peaks at 425 and 559 nm and a shoulder around 412 nm upon reduction with succinate, indicating a partial reduction of heme *b*, and peaks at 425, 525 and 558 nm upon the addition of dithionite.
(TIF)

Figure S2 **The three-dimensional homology model of trimeric** ***T. thermophilus*** **complex II (A) and visualization of trimer disruption upon placing a His-tag at the C-terminus of SdhB subunit (B).** The three-dimensional model of *T. thermophilus* complex II was built using the SWISS-MODEL server [1], [2], [3] and the structure of *E. coli* SQR (PDB ID: 1NEK) was used as the template. Complex II monomers are presented in blue, green and orange. The C-terminal arginine residue of each SdhB subunit is shown as magenta spheres while His_6_-tags are shown as red spheres.
1. Arnold K, Bordoli L, Kopp J, Schwede T (2006) The SWISS-MODEL workspace: a web-based environment for protein structure homology modelling. Bioinformatics 22: 195–201.
2. Kiefer F, Arnold K, Kunzli M, Bordoli L, Schwede T (2009) The SWISS-MODEL Repository and associated resources. Nucleic Acids Res 37: D387–D392.
3. Peitsch MC, Tschopp J (1995) Comparative molecular modelling of the Fas-ligand and other members of the TNF family. Mol Immunol 32: 761–772.
(TIF)

Figure S3 **The EPR spectra of the [2Fe-2S] cluster of the recombinant complex II from** ***T. thermophilus*** **reduced by a 10- (−) and 100- fold (–) excess of dithionite.** The vertical lines indicate the position of the *g*_x_ signal shifting from 2.021 to 2.022. Protein concentration was 10 µM. Spectra were recorded at a temperature of 50 K, microwave power of 64 mW and a modulation amplitude of 1 mT.
(TIF)

Figure S4 **Ligands of Fe-S cluster located within SdhB/FrdB subunit.** The sequences were aligned with MUSCLE (http://www.ebi.ac.uk/Tools/msa/muscle) [4] and rendered with Jalview with colouring at 85% identity [5] using the sequences from the respective crystal structures of avian SQR (PDB ID: 1YQ3), *E. coli* QFR (Ec_QFR; PDB ID: 1KF6), *E. coli* SQR (Ec_SQR; PDB ID: 1NEK), porcine SQR (PDB ID: 1ZOY) and *W. succinogenes* QFR (Ws_QFR; PDB ID: 2BS2). *T. thermophilus* SQR accession is RefSeq ID: YP_144719.1.
4. Edgar RC (2004) MUSCLE: multiple sequence alignment with high accuracy and high throughput. Nucleic Acids Res 32: 1792–1797.
5. Waterhouse AM, Procter JB, Martin DM, Clamp M, Barton GJ (2009) Jalview Version 2–a multiple sequence alignment editor and analysis workbench. Bioinformatics 25: 1189–1191.
(TIF)

Table S1 **Purification table of recombinantly-produced** ***T. thermophilus*** **complex II.**
(DOC)

Table S2 **Midpoint potentials of hemes** ***b*** **_H_ and** ***b*** **_L_ in different variants of complex II from** ***T. thermophilus.***
(DOC)
