# Supplementary figures and images for: Atypical Features of Thermus thermophilus Succinate:Quinone Reductase
Source: PLoS One. 2013 Jan 7;8(1):e53559. doi: 10.1371/journal.pone.0053559 (PMC3538594; doi:10.1371/journal.pone.0053559)

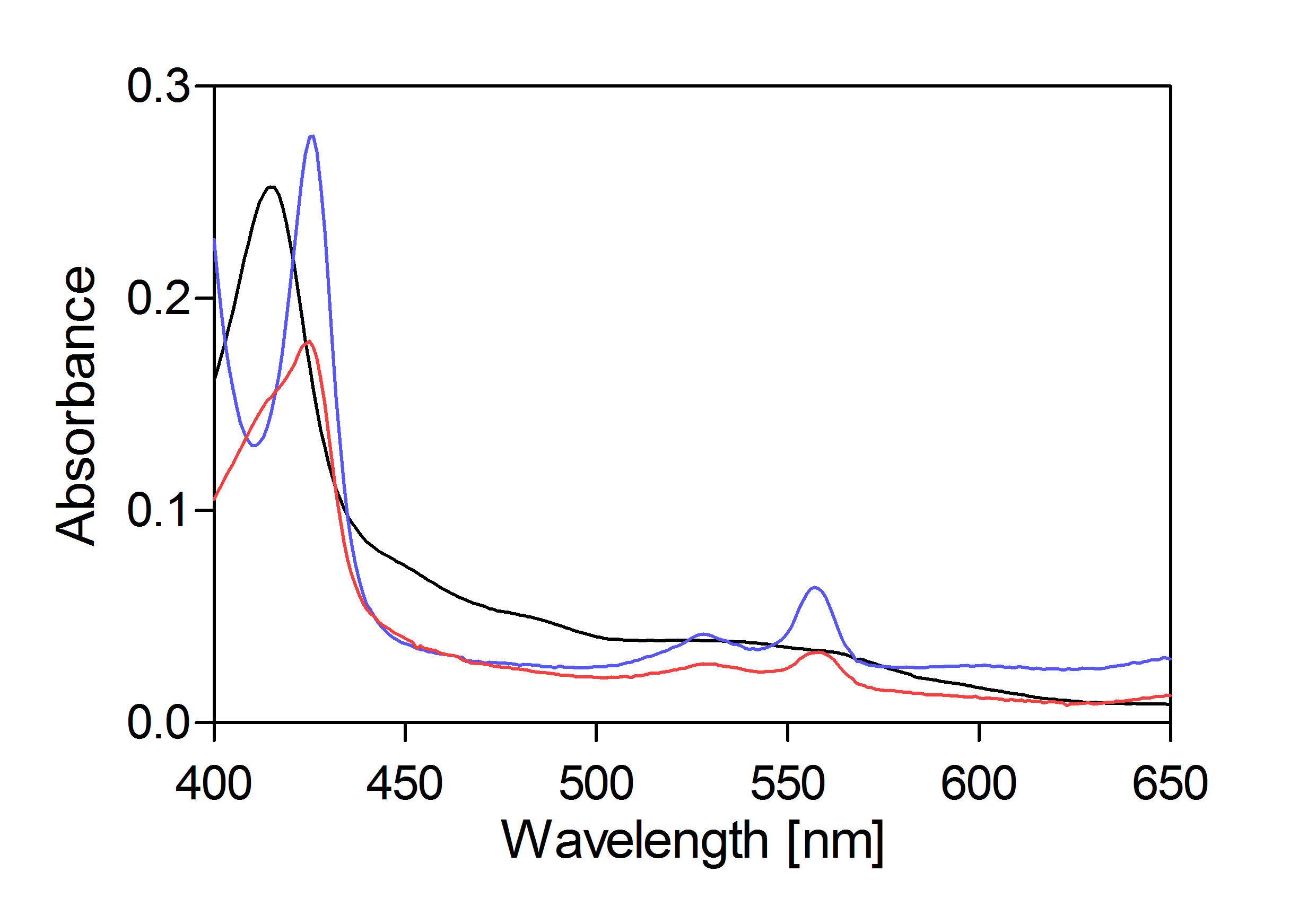

Supplement: Figure S1 — VIS spectra of recombinant complex II from T. thermophilus in its oxidized (black), succinate-reduced (red) and dithionite-reduced state (blue). All the recombinant variants of complex II exhibited the same spectroscopic features as the native enzyme with absorption peaks at 425 and 559 nm and a shoulder around 412 nm upon reduction with succinate, indicating a partial reduction of heme b, and peaks at 425, 525 and 558 nm upon the addition of dithionite. (TIF) [file pone.0053559.s001.tif]

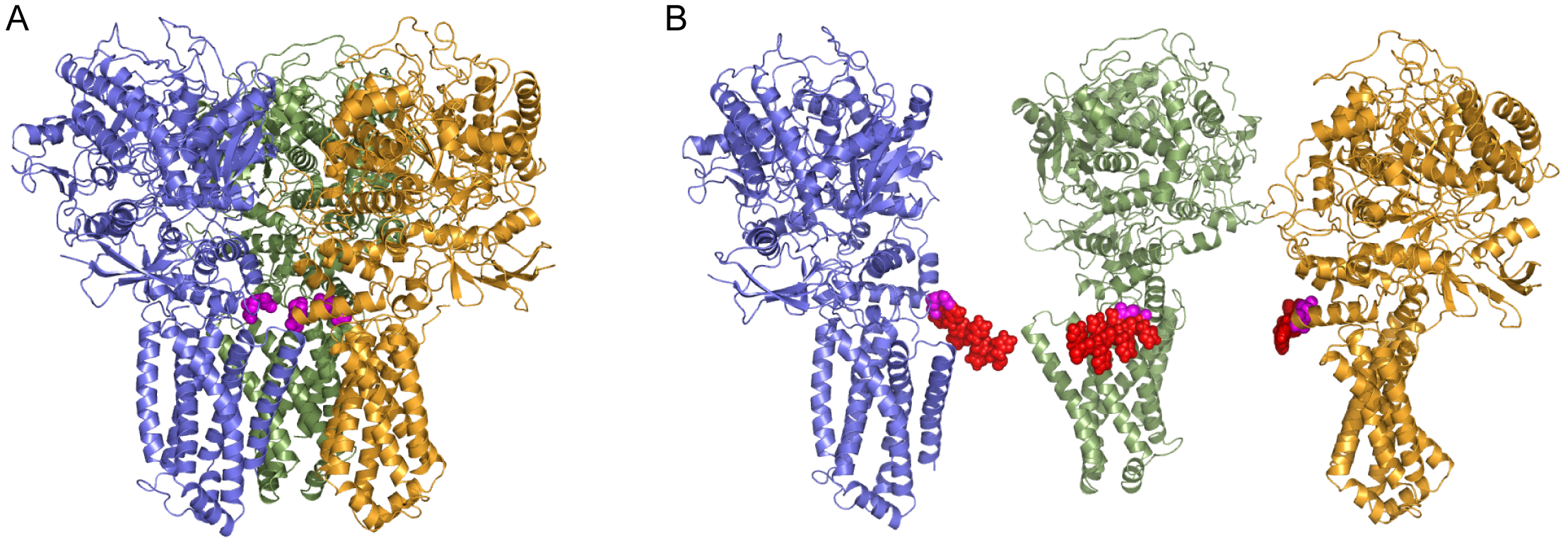

Supplement: Figure S2 — The three-dimensional homology model of trimeric T. thermophilus complex II (A) and visualization of trimer disruption upon placing a His-tag at the C-terminus of SdhB subunit (B). The three-dimensional model of T. thermophilus complex II was built using the SWISS-MODEL server [1], [2], [3] and the structure of E. coli SQR (PDB ID: 1NEK) was used as the template. Complex II monomers are presented in blue, green and orange. The C-terminal arginine residue of each SdhB subunit is shown as magenta spheres while His6-tags are shown as red spheres. 1. Arnold K, Bordoli L, Kopp J, Schwede T (2006) The SWISS-MODEL workspace: a web-based environment for protein structure homology modelling. Bioinformatics 22: 195–201. 2. Kiefer F, Arnold K, Kunzli M, Bordoli L, Schwede T (2009) The SWISS-MODEL Repository and associated resources. Nucleic Acids Res 37: D387–D392. 3. Peitsch MC, Tschopp J (1995) Comparative molecular modelling of the Fas-ligand and other members of the TNF family. Mol Immunol 32: 761–772. (TIF) [file pone.0053559.s002.tif]

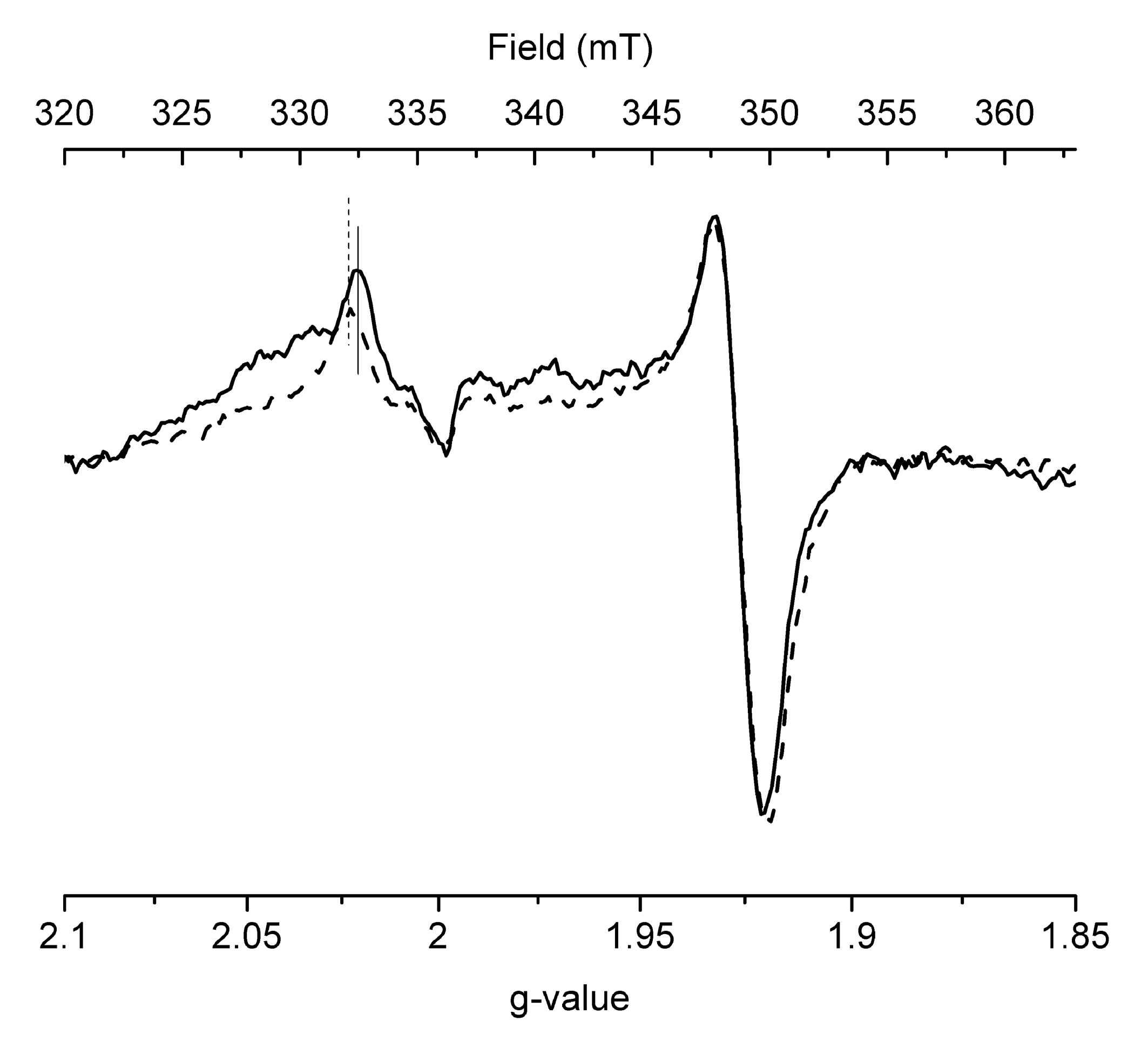

Supplement: Figure S3 — The EPR spectra of the [2Fe-2S] cluster of the recombinant complex II from T. thermophilus reduced by a 10- (−) and 100- fold (–) excess of dithionite. The vertical lines indicate the position of the g x signal shifting from 2.021 to 2.022. Protein concentration was 10 µM. Spectra were recorded at a temperature of 50 K, microwave power of 64 mW and a modulation amplitude of 1 mT. (TIF) [file pone.0053559.s003.tif]

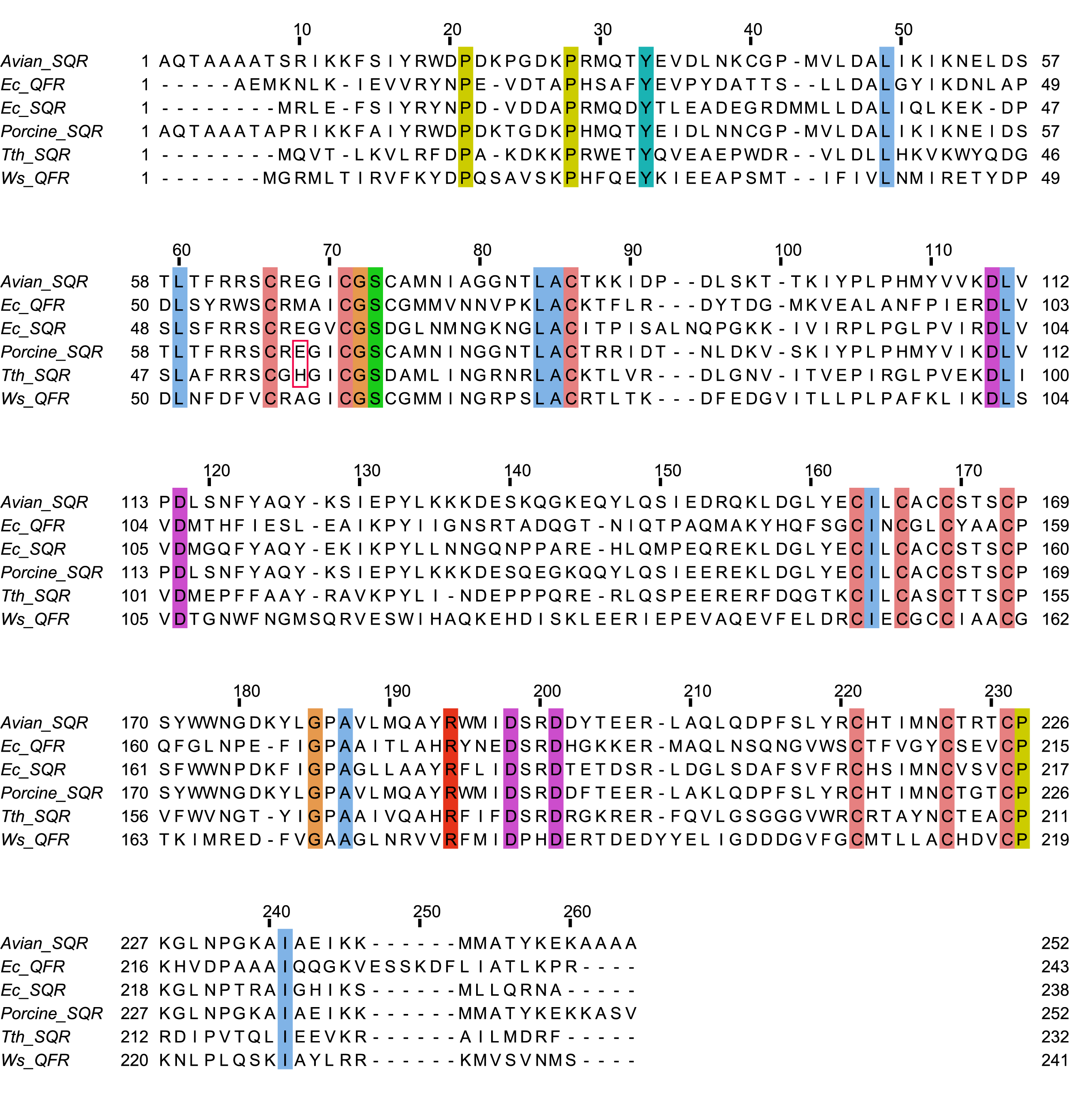

Supplement: Figure S4 — Ligands of Fe-S cluster located within SdhB/FrdB subunit. The sequences were aligned with MUSCLE (http://www.ebi.ac.uk/Tools/msa/muscle) [4] and rendered with Jalview with colouring at 85% identity [5] using the sequences from the respective crystal structures of avian SQR (PDB ID: 1YQ3), E. coli QFR (Ec_QFR; PDB ID: 1KF6), E. coli SQR (Ec_SQR; PDB ID: 1NEK), porcine SQR (PDB ID: 1ZOY) and W. succinogenes QFR (Ws_QFR; PDB ID: 2BS2). T. thermophilus SQR accession is RefSeq ID: YP_144719.1. 4. Edgar RC (2004) MUSCLE: multiple sequence alignment with high accuracy and high throughput. Nucleic Acids Res 32: 1792–1797. 5. Waterhouse AM, Procter JB, Martin DM, Clamp M, Barton GJ (2009) Jalview Version 2–a multiple sequence alignment editor and analysis workbench. Bioinformatics 25: 1189–1191. (TIF) [file pone.0053559.s004.tif]
