# Supplementary material for: Atypical Features of Thermus thermophilus Succinate:Quinone Reductase
Source: PLoS One. 2013 Jan 7;8(1):e53559. doi: 10.1371/journal.pone.0053559 (PMC3538594; doi:10.1371/journal.pone.0053559)
Supplement: Table S1 — Purification table of recombinantly-produced T. thermophilus complex II. (DOC) [file pone.0053559.s005.doc]

**Table S1. Purification table of recombinantly-produced *T. thermophilus* complex II.**

| **Purification step** | **total protein (mg)** | | | **Heme content (nmol)** | | | **heme/protein ratio (nmol/mg)** | | | **Yield (%)** | | |
| --- | --- | --- | --- | --- | --- | --- | --- | --- | --- | --- | --- | --- |
|  | **1** | **2** | **3** | **1** | **2** | **3** | **1** | **2** | **3** | **1** | **2** | **3** |
| **Membranes** | 195 | 245 | 290 | 1390 | 1650 | 1867 | 7.13 | 6.74 | 6.40 | 100 | 100 | 100 |
| **IMAC** | - | 44.5 | 46.7 | - | 612 | 652 | - | 13.75 | 14.00 | - | 37.1 | 34.9 |
| **TMAE** | 81 | - | - | 831 | - | - | 10.26 | - | - | 60 | - |  |
| **Gel filtration** | 66 | - | - | 767 | - | - | 11.62 | - | - | 55 | - |  |
| **Hydroxylapatite** | 49 | - | - | 656 | - | - | 13.39 | - | - | 47 | - |  |
| **TMAE** | 44 | 38 | 38.7 | 654 | 592 | 637 | 14.86 | 15.57 | 16.50 | 47 | 35.9 | 34.1 |
| **Gel filtration** | 37 | 35 | 34.7 | 608 | 581 | 678 | 16.43 | 16.60 | 16.60 | 44 | 35.2 | 30.9 |

*Values are given for 100g biomass*

**1** - wild type recombinant complex II (wt-rcII)

**2** - His-tagged recombinant complex II (rcII-SdhB-His_6_)

**3** - His-tagged recombinant complex II (rcII-His_8_-SdhB)
