# Supplementary material for: Atypical Features of Thermus thermophilus Succinate:Quinone Reductase
Source: PLoS One. 2013 Jan 7;8(1):e53559. doi: 10.1371/journal.pone.0053559 (PMC3538594; doi:10.1371/journal.pone.0053559)
Supplement: Table S2 — Midpoint potentials of hemes b H and b L in different variants of complex II from T. thermophilus. (DOC) [file pone.0053559.s006.doc]

**Table S2. Midpoint potentials of hemes *b*_H_ and *b*_L_ in different variants of complex II from *T. thermophilus.***

|  | **Heme midpoint potential** | |
| --- | --- | --- |
|  | ***b*_H_ (± 20 mV)** | ***b*_L_ (± 20 mV)** |
| **Native complex II** | - 20 | ‑ 160 |
| **wt-rcII** | + 5 | - 150 |
| **rcII-SdhB-His_6_** | + 10 | -170 |
| **rcII-His_8_-SdhB** | +10 | -215 |
